# Molecular assessment of bacterial vaginosis by *Lactobacillus* abundance and species diversity

**DOI:** 10.1186/s12879-016-1513-3

**Published:** 2016-04-23

**Authors:** Joke A. M. Dols, Douwe Molenaar, Jannie J. van der Helm, Martien P. M. Caspers, Alie de Kat Angelino-Bart, Frank H. J. Schuren, Adrianus G. C. L. Speksnijder, Hans V. Westerhoff, Jan Hendrik Richardus, Mathilde E. Boon, Gregor Reid, Henry J. C. de Vries, Remco Kort

**Affiliations:** Molecular Cell Physiology, Faculty of Earth and Life Sciences, VU University, Amsterdam, The Netherlands; Department of Public Health, Erasmus MC, University Medical Center Rotterdam, Rotterdam, The Netherlands; STI Outpatient Clinic, Public Health Service of Amsterdam (GGD Amsterdam), Amsterdam, The Netherlands; Netherlands Organisation for Applied Scientific Research (TNO), Microbiology and Systems Biology, Utrechtseweg 48, 3704HE Zeist, The Netherlands; Naturalis Biodiversity Center, Darwinweg 2, Leiden, The Netherlands; Synthetic Systems Biology, Swammerdam Institute for Life Sciences, University of Amsterdam, Amsterdam, The Netherlands; Manchester Centre for Integrative Systems Biology, University of Manchester, Manchester, UK; Leiden Cytology and Pathology Laboratory, Leiden, The Netherlands; Canadian Center for Human Microbiome and Probiotic Research, Lawson Health Research Institute, London, Ontorio, Canada; Department of Microbiology and Immunology, Division of Urology, Department of Surgery, Western University, London, Ontario Canada; Amsterdam Centre for Infection and Immunity (CINIMA), Academic Medical Center (AMC), University of Amsterdam, Amsterdam, The Netherlands; Department of Dermatology, Academic Medical Center (AMC), University of Amsterdam, Amsterdam, The Netherlands; Micropia, Natura Artis Magistra, Plantage Kerklaan 38-40, 1018 CZ Amsterdam, The Netherlands; Present address: Achter de Hor 2, 9304 TN Lieveren, The Netherlands

**Keywords:** Vaginal microbiota, Bacterial vaginosis, Nucleotide-based microarrays, 16S rRNA amplicon sequencing, IS-profiling, *Lactobacillus crispatus*, *Lactobacillus iners*, *Gardnerella vaginalis*, Gini-Simpson index

## Abstract

**Background:**

To date, women are most often diagnosed with bacterial vaginosis (BV) using microscopy based Nugent scoring or Amsel criteria. However, the accuracy is less than optimal. The aim of the present study was to confirm the identity of known BV-associated composition profiles and evaluate indicators for BV using three molecular methods.

**Methods:**

Evaluation of indicators for BV was carried out by 16S rRNA amplicon sequencing of the V5-V7 region, a tailor-made 16S rRNA oligonucleotide-based microarray, and a PCR-based profiling technique termed IS-profiling, which is based on fragment variability of the 16S-23S rRNA intergenic spacer region. An inventory of vaginal bacterial species was obtained from 40 females attending a Dutch sexually transmitted infection outpatient clinic, of which 20 diagnosed with BV (Nugent score 7–10), and 20 BV negative (Nugent score 0–3).

**Results:**

Analysis of the bacterial communities by 16S rRNA amplicon sequencing revealed two clusters in the BV negative women, dominated by either *Lactobacillus iners* or *Lactobacillus crispatus* and three distinct clusters in the BV positive women. In the former, there was a virtually complete, negative correlation between *L. crispatus* and *L. iners*. BV positive subjects showed cluster profiles that were relatively high in bacterial species diversity and dominated by anaerobic species, including *Gardnerella vaginalis,* and those belonging to the Families of *Lachnospiraceae* and *Leptotrichiaceae*. Accordingly, the Gini-Simpson index of species diversity, and the relative abundance *Lactobacillus* species appeared consistent indicators for BV. Under the conditions used, only the 16S rRNA amplicon sequencing method was suitable to assess species diversity, while all three molecular composition profiling methods were able to indicate *Lactobacillus* abundance in the vaginal microbiota.

**Conclusion:**

An affordable and simple molecular test showing a depletion of the genus *Lactobacillus* in combination with an increased species diversity of vaginal microbiota could serve as an alternative and practical diagnostic method for the assessment of BV.

**Electronic supplementary material:**

The online version of this article (doi:10.1186/s12879-016-1513-3) contains supplementary material, which is available to authorized users.

## Background

Bacterial vaginosis (BV) is an aberrant state of the vaginal microbiota, which is characterized by a depletion of lactobacilli, an increased diversity of the bacterial population and an elevated pH. It is one the most common vaginal syndromes in fertile, premenopausal and pregnant women [1]. Vaginal malodor is a common reason for women to consult a physician, but BV can also occur without malodor or other symptoms and signs. The association of BV with pre-term birth [2] and increased risk of sexually transmitted infections (STIs) [3] makes it important that a correct diagnosis is made. To date, treatment failure rates and recurrence rates of BV remain high [4, 5].

Diagnoses based on the Amsel criteria [6], and microscopic methods, such as the Nugent score [7], have their limitations. A study we performed on samples from South African women showed low specificity when diagnosis of BV was based upon *Gardnerella vaginalis* for the Nugent score. In 24 % of women who were BV-negative, *Gardnerella* was present [8]. Another limitations is inter-observer variation of microscopic slides. Therefore, new molecular methodologies have been used recently to study the vaginal microbiome. Next generation sequencing (NGS) technologies, such as Illumina and 454-sequencing, can detect known and unknown sequences without prior knowledge of the species in the sample, facilitating in-depth analysis of microbial community diversity [9]. As a result of the rapid development of sequencing platforms, they have become more affordable and accurate.

Microarray analysis [8, 10] requires pre-selection of the organisms expected in the samples and is subject to cross-hybridization between highly similar sequences [11]. However, this method has been a useful tool to detect BV-associated species, including *Gardnerella vaginalis*, *Atopobium vaginae, Dialister* species, *Megasphaera* species, *Mobiluncus mulieris, Sneathia sanguinegens,* and *Prevotella* species [8, 10]. The method gave comparable results to the organisms detected using Illumina 16S rRNA amplicon sequencing on Tanzanian BV subjects [12]. A third, relatively fast method, which has not been applied previously to determine the composition of the vaginal microbiota, is based on the profiling of the 16S–23S rRNA intergenic spacer (IS)-regions and has been called IS-profiling [13].

As there is a strong need to develop a new gold standard for BV, it is important establish universal markers based on molecular methods. Therefore, we first evaluated the outcome of 16S rRNA amplicon sequencing of the vaginal microbiota of women positive and negative for BV on the basis of conventional Nugent scores in order to confirm the composition of bacterial populations known to be associated with BV [14]. Second, we selected molecular indicators for BV, including overall abundance of the genus *Lactobacillus* and species diversity, followed by evaluation of the indicators with three molecular methods, high-throughput 16S rRNA amplicon sequencing, oligonucleotide-based microarrays, and IS-profiling. The results of this study pave the way for further development of a universal, PCR-based molecular diagnostic test for BV.

## Methods

### Subjects and sampling

For women with high-risk for STIs, routine screening is offered at the STI outpatient clinic of the Public Health Service Amsterdam (GGD, Amsterdam). The subjects included in this study had either vaginal signs and/or symptoms, were referred by a physician for STI testing or tested because a sexual partner with a proven STI had notified them. Samples were collected in June and July 2012. A standard cervical examination was performed and a cotton swab was used to remove abundant mucus prior to the collection of a sample for *Chlamydia trachomatis* and *Neisseria gonorrhoeae* screening. The swab to remove abundant mucus is not normally used for routine testing and is discarded. However, for this study the cotton swab was re-suspended in screw-cap coded tubes with Amies transport media to which additional 15 % glycerol and cysteïne solution had been added. Immediately, the tubes were placed in liquid nitrogen and stored at −80 °C. Samples did not disclose any subject names or other patient identification (date of birth, patient file number etc.), and were sent on dry ice to the Netherlands Organisation for Applied Scientific Research (TNO) for further analysis. We studied the vaginal microbiota of 40 subjects, of which 20 BV-negative and 20 BV-positive, by selection of low (0–3) and high (7–10) Nugent scores, respectively.

### STI status

STI-screening consisted of HIV status (determined by serology), syphilis (serology and/or nucleic acid amplification test (NAAT), gonorrhea (culture and/or NAAT), *Chlamydia* (urogenital, anogenital and throat region; NAAT), herpes type 1 and 2 (PCR), *Trichomonas* (PCR), hepatitis B (serology), *Moluscum contagiosum* (clinical appearance), scabies (clinical appearance), ulcus (clinical appearance), and pelvic inflammatory disease (PID; clinical appearance).

### Diagnosis of BV and vulvovaginal candidiasis

To determine if women met the criteria for BV or had vulvovaginal candidiasis (VVC), microscopic scoring was performed (potassium hydroxide (KOH) preparation and Gram-stain). A vaginal smear was examined using the Nugent scale [7], which includes the scores 0–3 as Normal; 4–6, Intermediate; and 7–10 for BV. The diagnosis of BV was based on the Nugent Gram stain and the presence of three Amsel criteria [6], characteristic vaginal discharge, clue cells, and positive amine test. Measurement of the pH was not part of the routine screening procedure.

### DNA isolation

The DNA isolations were performed as described in detail by Zhao and others [15]. Briefly, samples were mixed with 150 μl Agowa lysis buffer BL, 350 μl zirconium beads (0.1 mm; suspended in milli Q-water), and 200 μl phenol and lysed in a BeadBeater (BioSpec Products, Bartlesville) for 2 min. The aqueous phase was collected after spinning (5 min ~9.000 g) and DNA was isolated via Agowa binding beads.

### High-throughput sequencing and taxonomic classification

Sequence analysis was performed on a 454 GS-FLX-Titanium Sequencer (Life Sciences (Roche), Branford, CT) as described previously [16]. The amount of bacterial template in the isolated DNA samples was determined with a universal quantitative PCR for 16S rRNA gene [17]. Afterwards, a 16S rRNA gene amplicon library spanning variable regions V5-V7 was generated [16]. FASTA-formatted sequences and corresponding quality scores were extracted from the data file generated by the GS-FLX Titanium sequencer using the GS Amplicon software package (Roche, Branford, CT) and processed using modules from the Mothur v. 1.22.2 software platform [18].

On average 2745 (minimum 96, maximum 5932, standard deviation 1304) sequence reads were generated for the total of 40 amplified DNA fragments. Only one DNA sample (TCMID116) resulted insufficient reads (less than 100) and was excluded from subsequent analyses. Sequences were de-noised using a pseudo-single linkage algorithm with the goal of removing sequences that are likely pyrosequencing errors using the “pre.cluster” command [19]. Potentially chimeric sequences were detected and removed using the “chimera.uchime” command [20]. Fragments were aligned and a consensus was made for identification purposes.

The family, genus and species determination is based on the comparison of the specific probe sequence with sequences derived from type strains in the RDP database [21]. A sequence matched with the RDP database with a homology score of 1 is named after the species, if possible. A homology score <1 is named after the genus or family name. Names of certain species, genera or families which are presented more than once, result from a difference on sequence level. The amplicon sequences, numbers of reads for each sequence, and taxonomic classifications have been presented in Additional file 1.

### Statistical analysis of 16S rRNA amplicon sequences

The Pearson correlation coefficient of counts of all pairs of unique sequences from the 40 samples (585 in total) were calculated (leading to a 585 by 585 matrix of correlation coefficients). Some sequences were highly positively correlated, having correlation coefficients close to or equal to 1, namely those that occurred in only 1 sample, and those that most likely originate from the same species. The complexity of the data was reduced by binning the counts of such highly correlated sequences to one new sequence count variable. The total number of new variables (unique sequences and sets of highly correlated sequences) was 149. The lower boundary used for binning counts of correlated sequences was a Pearson correlation coefficient of 0.95. The resulting data were clustered. Data were normalized by calculating relative numbers per sample. The square root of these values was taken. The Kruskal-Wallis test (a robust variant of one-way ANOVA), was performed on the relation between relative species abundance and the five main sample clusters.

Using random forest analysis, correlations between relative abundances of bacterial sequences and the clinical variables with a moderate variability were examined by calculating a “Node Purity value” (IncNodePurity). The mean increase in node purity is a measure of how each variable, the sequence abundance of a specific species, genus or family, contributes to the classification of the sample by minimizing the residual sum of squares in regression.

### Nucleotide-based microarray analysis

Literature research and sequence analysis of a number of vaginal microbiome samples were used for the design of a tailor-made nucleotide-based microarray [8, 22]. Taxonomic selection of vaginal bacterial species for the microarray was expanded based on denaturing gradient gel electrophoresis (DGGE) analysis (data not shown). For each bacterial species represented on the microarray, one or more unique short oligonucleotide sequences from within the 16 s rRNA gene were selected [8, 22]. The microarray data, based on consistent signal to background ratios of fluorescence intensity after hybridization, have been indicated for each selected oligonucleotide probe in Additional file 2.

### IS-profiling

A PCR-based profiling technique for high-throughput analysis of the microbiome was performed as described by Budding and others [23]. The amplification of the IS-regions was performed with the IS-pro assay (IS-Diagnostics, Amsterdam, the Netherlands). The IS-pro method involves bacterial species differentiation by the length of the 16S-23S rRNA intergenic spacer region with taxonomic classification by phylum-specific fluorescent labeling of PCR primers. The IS-pro procedure includes PCR’s for the phyla Firmicutes, Actinobacteria, Fusobacteria, and Verrucomicrobia (FAFV), Bacteroidetes and Proteobacteria [23, 24]. The IS-profiling was performed on vaginal microbiota samples selected from each of five clusters identified in this study. A total of 15 samples were analyzed by IS-profiling, including representatives of BV-negative cluster I (TCMID 101, 103, 113), and cluster II (TCMID 102, 111, 112), as well as BV-positive cluster III (TCMID 126, 135), cluster IV (TCMID 134, 136), and cluster V (TCMID 117, 118, 120). As a control, IS-pro analyses was performed on a *L. crispatus* strain isolated from TCMID 103 (BV-negative, Nugent score 0), and on a *L. iners* strain, isolated from TCMID 134 (BV-positive, Nugent score 10).

### Comparative analysis of molecular markers for bacterial vaginosis

Species diversity based on next generation sequencing, microarray and IS-profiling has been calculated by the Gini-Simpson index [25]. The Gini-Simpson index measures the degree a sequence abundance contributes to the classification of the sample. Each microarray probe was scored by counting only positive detection, *i.e.* having a value above the detection threshold (≥5 signal-to-background ratio). Relative abundance is calculated by selecting all representing probes and calculating the number of percent of a species of a particular kind relative to the total number of species per sample. The read-out of the IS-profiling was analyzed by the mean log2 intensity in Relative Fluorescence Units (RFU) per phylum for BV positive clusters and BV negative clusters. Analysis was conducted using the software environment for statistical computing and graphics R version 3.2.2 [26] and the MeV microarray software suite version 4.9 [27].

## Results

### Bacterial vaginosis and STI-status

A total of 40 cervical swab samples were collected from women older than 18 years of age. The bacterial populations in these samples were analyzed from the first twenty consecutive patients with a BV-positive score (Nugent scale 7–10), and the first 20 consecutive patients with a BV-negative score (Nugent scale 0–3). The latterincluded 18 (45 %) of European or Asian origin and 2 (5 %) of a Hispanic background, with the remainder Caucasian. The BV-positive women comprised 9 (23 %) of European or Asian origin versus 11 (28 %) of a Hispanic background, (Additional file 3). Characteristics of the women and STI-status are shown in Table 1. According to the Amsel criteria, 21 women were positive for BV, with only one discrepancy between the Amsel and Nugent method. Two subjects (5 %) tested positive for VVC. An STI was detected in 10 women (25 %), with *C. trachomatis* being the most prevalent (18 %). None of the women had HIV, Syphilis, Gonorrhoea, Hepatitis B, *Moluscum contagiosum*, Scabies, genito-ulcerative disease, or PID.

**Table 1:** Patient characteristics and STI status of 20 women with BV and 20 women without BV, as diagnosed by the STI outpatient clinic Public Health Service Amsterdam, in June and July 2012

		BV-negative women *n* = 20 (% of BV-negative women)	BV-positive women *n* = 20 (% of BV-positive women)
Inclusion criteria			
Referred by physician or notified by sexual partner		4 (20 %)	4 (20 %)
Vaginal complaints		16 (80 %)	20 (100 %)
Patient characteristics			
Median age (years) (IQR)		23 (22–25)	22.5 (21–27)
Any STI		3 (15 %)	7 (35 %)
Chlamydia		2 (10 %)	5 (25 %)
Herpes	Type 1	1 (5 %)	0
	Type 2	0	0
Condyloma		1 (5 %)	1 (5 %)
Trichomoniasis		0	1 (5 %)
HIV, Syphilis, Gonorrhoea, Hepatitis B, Moluscum Contagiosum, Scabies, Ulcus, PID		All negative	

The subjects included in this study had either vaginal signs and/or symptoms, were referred by a physician for STI testing or tested because a sexual partner with a proven STI had notified them. The number of women based on the inclusion criteria and STI status is shown (*n*). The percentage (%) of women with STIs is shown

### Identification of five distinct vaginal microbiota types

**Fig. 1:**
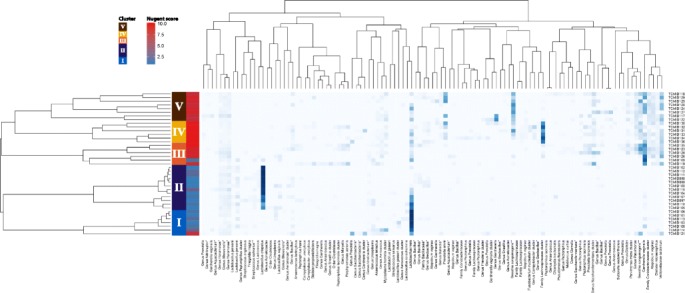
Cluster analysis of next generation sequencing data of women without and with various extents of BV. At the top the first color bar from the left shows the numbering and color-coding of the grouping that the clustering of the women (the dimension of the abscissa) suggests. The second color bar refers to the low (*blue*) to high (*red*) Nugent score of the women. On the abscissa the family, genus, species identities are shown clustered on the basis of this next generation sequencing data set. An asterisk indicates the species that are considered to be a result of isolation of background DNA from bacterial species of a non-vaginal origin, as based on systematic comparisons with previously reported compositions of the vaginal microbiome [22, 30]. Two asterisks indicate multiple hits with identical similarity in RDP database: the species *Streptococcus salivarius*, which cannot be discriminated from *Enterococcus faecium* and *Sneathia sanguinegens,* which cannot be discriminated from *Leptotrichia amnionii*

Analysis of the 16S rRNA amplicon sequencing data (Fig. 1) revealed five distinct types or clusters with two subgroups perhaps within the fifth type. Three clusters were identified in the BV-positive subjects and two in the BV-negative samples (Nugent score ≤ 3). Sample TCMID 121, positive for BV according to the Nugent criteria, was classified as cluster I. Apart from a high abundance of *L. iners*, there was a high abundance of *Prevotella*, *G. vaginalis*, and *Sneathia sanguinegens* in this sample.

Results of the Kruskal-Wallis test, that determined which species distribution differentiated most significantly between clusters, are shown in Table 2. Clearly, in BV-negative samples, a relatively low species diversity is observed, with the microbiome dominated by either *L. crispatus* or *L. iners*. Cluster I was characterized by *L. iners* at 81 %, while cluster II was dominated by *L. crispatus* at 79 % with *L. iners* present at 17 %. Cluster III comprised a group of mainly BV-positive women (5 out of 6), with the vaginal microbiome dominated by *G. vaginalis* (43 %) and *Leptotrichia amnionii* (12 %). Sample TCMID 109 scored negative for BV according to the Nugent criteria (score 1), however according to the Amsel score this sample was BV-positive (vaginal discharge clue cells, and positive amine test; in combination with pseudohyphae). The analysis of 16S rRNA amplicon sequences indicated a high abundance of *G. vaginalis* in this sample, thereby classifying it as cluster III. Sequences of the Family Lachnospiraceae were the most abundant in cluster IV (52 %). Cluster V could be identified as the most diverse cluster, including bacterial species at similar abundance, including *Sn. sanguinegens* (22 %) and *G. vaginalis* (15 %).

**Table 2:** The relation between species abundance and the five main sample clusters using the Kruskal Wallis test

Species	K-W rank sum	Cl. I (%)	Cl. II (%)	Cl. III (%)	Cl. IV (%)	Cl. V (%)
		BV neg.	BV neg.	BV pos.	BV pos.	BV pos.
*Lactobacillus crispatus*	33	0	**79**	0.4	0	0
*Sneathia sanguinegens* ^*a*^	30	0	0	0.2	0.8	**22**
Coriobacteriaceae	28	0	0	1.3	0.4	2.0
*Dialister micraerophilus*	26	0.1	0	0.5	0.2	0.4
*Atopobium vaginae*	25	0	0	1.7	1.0	1.8
Veillonellaceae	23	1.3	0	11	5.8	12
*Parvimonas sp*	22	2	0	2.2	0.9	1.7
*Saccharofermentans*	22	0	0	6.6	0.8	2.1
*Leptotrichia amnionii* ^*a*^	21	2.6	0	12	4.9	6.6
*Gardnerella vaginalis*	21	1.3	0.1	**43**	4.1	15
Lachnospiraceae	21	0.1	0	0.4	**52**	0.1
*Prevotella amnii*	18	0	0	0	6.5	13
*Campylobacter sp*	16	0	0	0	1.5	0
*Lactobaccillus iners*	16	**81**	17	5.4	5.5	5.5
*Peptoniphilus lacrimalis*	15	0	0	0.2	0.1	0
*Lactobacillus jensenii*	15	0.5	0.7	0.3	0	0
*Dialister sp*	15	0	0	1.1	0.1	0.7

Per cluster the abundance of the species percentage is shown (*p* ≤ 0.02). The *p*-value is defined as the probability of observing a K-W rank sum of the size reported or more extreme when the null hypothesis is true (null hypothesis is that the distribution equal over all clusters for the selected species or bacterial Family). The K-W rank sum expresses the deviation from the distribution under the null hypothesis. For each cluster the most dominant species was printed in boldface. Values expressed as percentage and were rounded to two significant digits

^a^ *Sneathia sanguinegens* could not be unambiguously discriminated from *Leptotrichia amnionii*

**Table 3:** Comparative analysis of indicators for bacterial vaginosis assessed with the three molecular methods 16S rRNA amplicon sequencing, microarray, and IS profiling

Cl.	TCMID	BV	Nugent score	Gini Simpson index			Relative abundance genus *Lactobacillus*			Relative abundance *L. crispatus*			Relative abundance *L. iners*		
				Seq	Micr	IS-pro	Seq	Micr	IS-pro	Seq	Micr	IS-pro	Seq	Micr	IS-pro
I	101	BV-	0	0.03	0.73	0.69	0.98	0.89	0.98	0.00	0.00	0.02	0.98	0.46	0.95
I	103	BV-	0	0.12	0.82	0.34	0.99	0.91	0.92	0.00	0.00	0.00	0.93	0.53	0.92
I	113	BV-	2	0.09	0.87	0.80	0.97	0.49	0.95	0.00	0.00	0.01	0.95	0.22	0.69
II	111	BV-	2	0.05	0.61	0.60	0.97	0.73	0.92	0.97	0.53	0.92	0.00	0.00	0.00
II	102	BV-	0	0.05	0.67	0.67	0.99	0.98	0.97	0.97	0.69	0.89	0.01	0.00	0.06
II	112	BV-	2	0.01	0.74	0.67	1.00	1.00	0.97	0.99	0.65	0.94	0.00	0.00	0.00
III	126	BV+	9	0.60	0.78	0.81	0.05	0.00	0.32	0.00	0.00	0.00	0.04	0.00	0.32
III	135	BV+	10	0.77	0.89	0.86	0.05	0.01	0.18	0.00	0.00	0.00	0.05	0.01	0.18
IV	134	BV+	10	0.44	0.91	0.97	0.01	0.00	0.10	0.00	0.00	0.00	0.01	0.00	0.10
IV	136	BV+	10	0.68	0.85	0.87	0.13	0.11	0.26	0.00	0.00	0.00	0.13	0.03	0.26
V	117	BV+	8	0.82	0.82	0.86	0.01	0.00	0.04	0.00	0.00	0.00	0.01	0.00	0.04
V	118	BV+	8	0.86	0.87	0.85	0.02	0.00	0.16	0.00	0.00	0.00	0.02	0.00	0.16
V	120	BV+	8	0.76	0.87	0.83	0.05	0.00	0.13	0.00	0.00	0.00	0.05	0.00	0.13

The indicators, Gini-Simpson index, abundance of the genus *Lactobacillus*, and *L. crispatus*, and *L. iners* have been shown for representatives of each of the 5 clusters (a total of 13 samples). The Gini-Simpson index represents the species diversity (0 = low diversity, 1 = high diversity), the abundance of the genus *Lactobacillus*, and *L. crispatus*, and *L. iners* is shown as a relative abundance between 0 and 1

### Characteristics of vaginal microbiota compositions

**Fig. 2:**
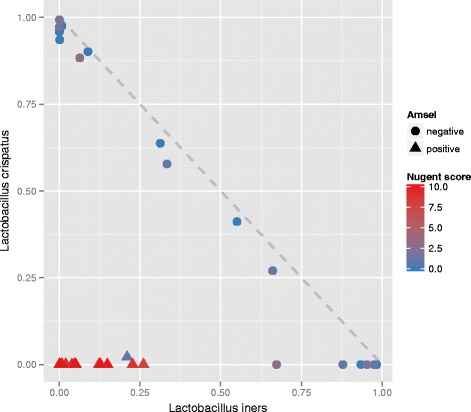
Correlation between *Lactobacillus crispatus* and *Lactobacillus iners* based on 16S rRNA amplicon sequencing. The abscissa and ordinate show, respectively, the fractions of *L. iners* and *L. crispatus* sequences relative to the total number of sequences. The color bar shows the color code: low (*blue*) to high (*red*) Nugent score of the women

**Fig. 3:**
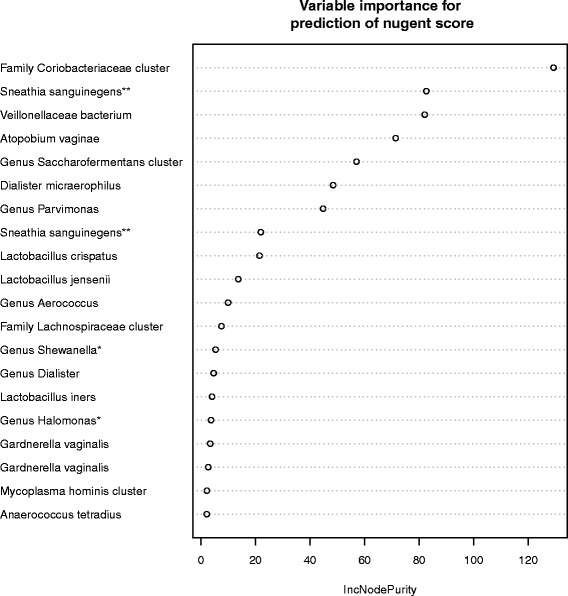
Predictive power of various microbial species for BV. The abscissa shows the mean “increase in node purity” for the prediction of the Nugent score (a measure of how the sequence abundance of the specific species or family denoted on the ordinate contributes to the classification of the sample). The ordinate presents the family, genus, species determination on the basis of 16S rRNA amplicon sequencing. Two different strains of G. vaginalis are presented

The correlation between *L. crispatus* and *L. iners* abundance across the women is indicated in Fig. 2. In the BV-negative subjects, there was a negative correlation of almost one between *L. crispatus* and *L. iners*. These women had a high abundance of *L. crispatus* or *L. iners*. All women with BV, and only 37 % of the women without BV were deficient of *L. crispatus*. The power of various 16S rRNA amplicon sequence based identifiers for the prediction of the Nugent-score is shown using the Node Purity value (Fig. 3). The strongest correlation between sequence abundance and Nugent score was found with sequences belonging to non-lactic acid bacteria, including the Family of the Coriobacteriaceae (including *A. vaginae*)*,* Leptotrichiaceae (*Sn. sanguinegens* or *L. amnionii*)*,* and of Veillonellaceae (including *Dialister micraerophilus*). The presence of sequences belonging to the Family of the Coriobacteriaceae was the most discriminating, comparing the two BV negative clusters I and II with the three BV positive clusters III, IV and V (see Fig. 3). There was no correlation between the sequence abundances and other clinical variables, including STI status, VVC, age, or symptoms and signs reported by patients. A larger sample size may be needed to detect correlations between these variables and vaginal microbiota composition.

**Fig. 4:**
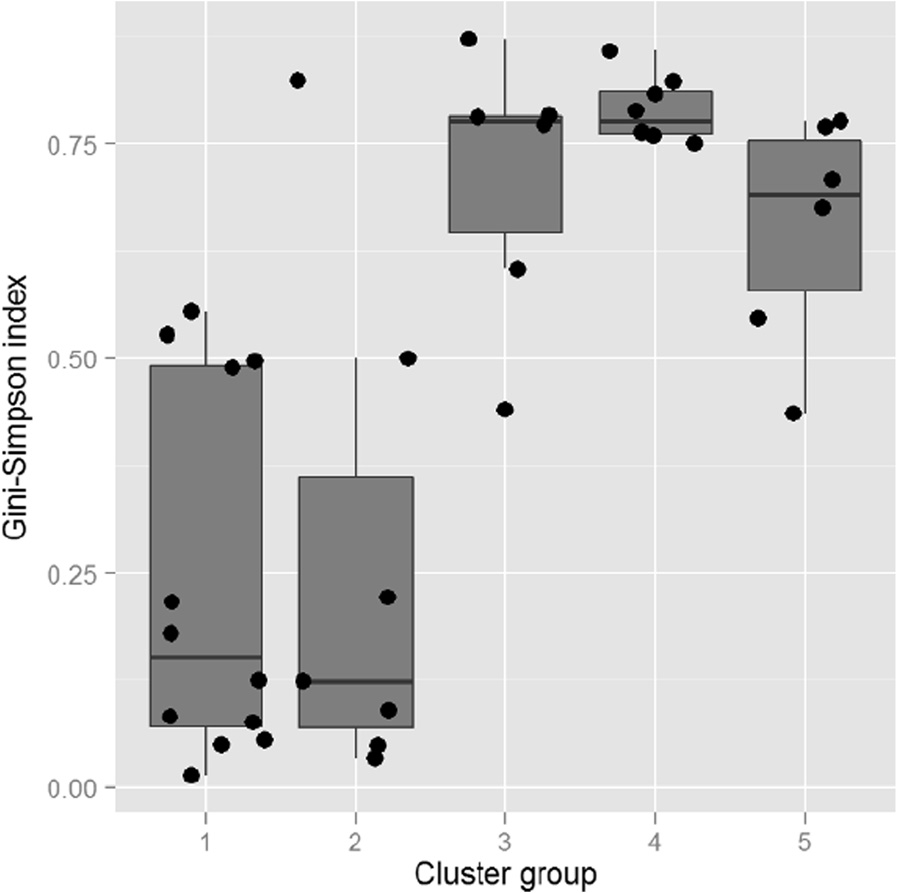
A boxplot relating species diversity based on 16S rRNA amplicon sequencing to BV. Individuals were first classified in clusters shown in sequence of increasing BV diagnosis on the abscissa. The dots give the Gini-Simpson index for individuals in different clusters. The boxes represent the distributions of the Gini-Simpson index and show its median and interquartile range (IQR) for each cluster. Whiskers extend to the furthest data point that is within 1.5 times the IQR

The bacterial species diversity of the various clusters is based on the 16S rRNA amplicon sequences and expressed as the Gini-Simpson index (Fig. 4). In the clusters of BV positive women (cluster III, cluster IV and cluster V) the microbiome showed a higher diversity of species than in the clusters of BV negative women based on the Gini-Simpson index (cluster I and cluster II), although there were a few individual exceptions.

### Microarray-based bacterial vaginosis profiles

**Fig. 5:**
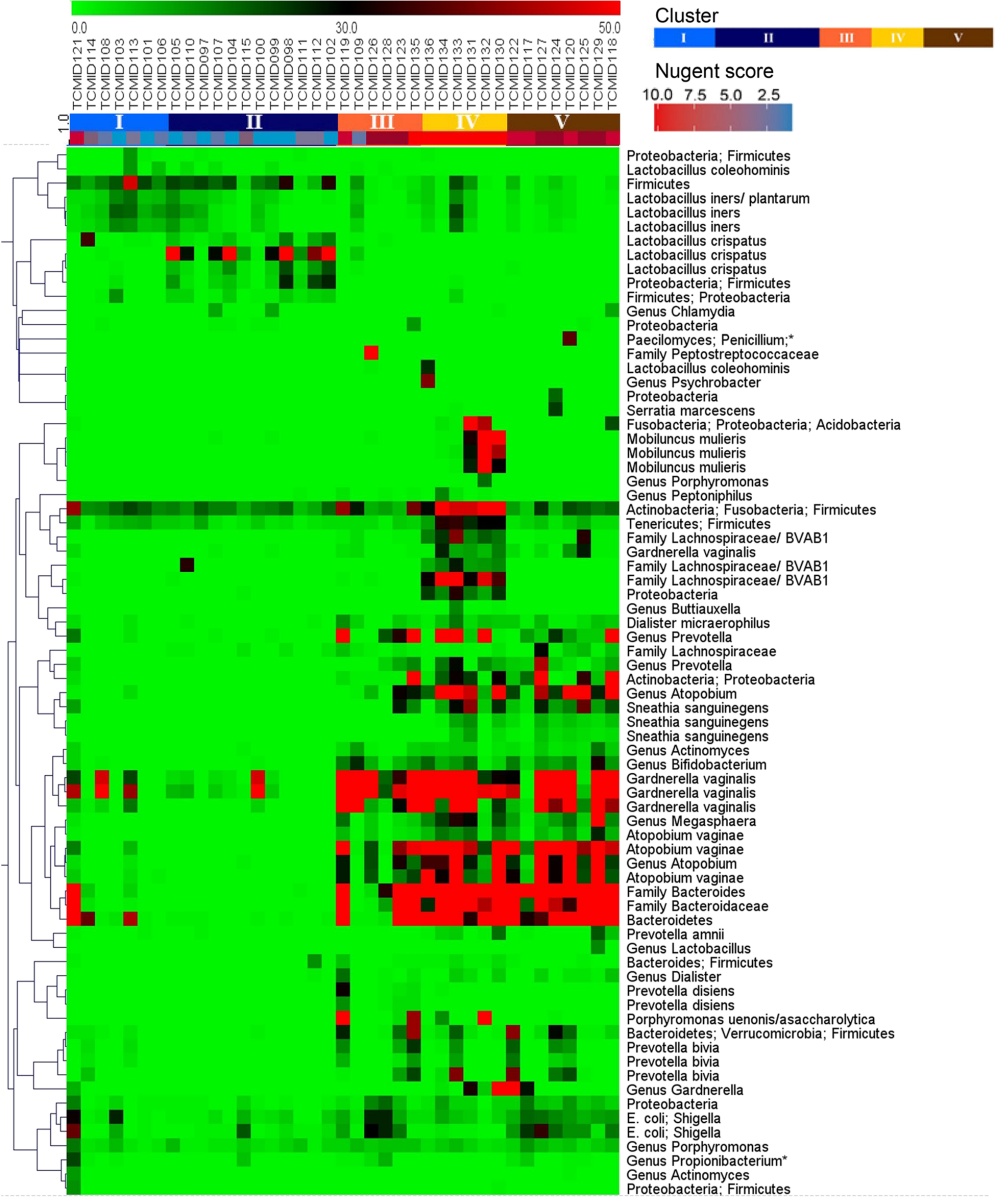
Organism clustering on the basis of microarray analysis, plotted versus clustering on the basis of the 16S rRNA amplicon sequencing. In the middle, the uppermost color bar represents the color coding of the fluorescence intensity (arbitrary units, numbers 0.0–50.0) of DNA hybridizations, the middle color bar shows the cluster the individual was classified into and the lower color bar shows the low (*blue*) to high (*red*) Nugent score of the individuals. On the right, the upper color bar shows the clustering color code and the lower color bar the Nugent score color code

The cluster analysis of microarray results is shown in Fig. 5. The abscissa orders the women on the basis of their cluster defined by the previous cluster analysis of 16S rRNA amplicon sequencing. Firmicutes, mainly Lactobacillaceae, were more present in BV negative than in BV positive clusters. Bacteroidetes abounded in the BV positive clusters. Cluster II, again (Table 2) identified by the presence of *L. crispatus*, and cluster IV, again identified by the presence of Family Lachnospiraceae, could also be distinguished. A separate cluster III marked by *G. vaginalis* or *Sn. sanguinegens* (Table 2) could not be distinguished in the microarray profiles.

### Bacterial vaginosis profiles by IS-profiling

**Fig. 6:**
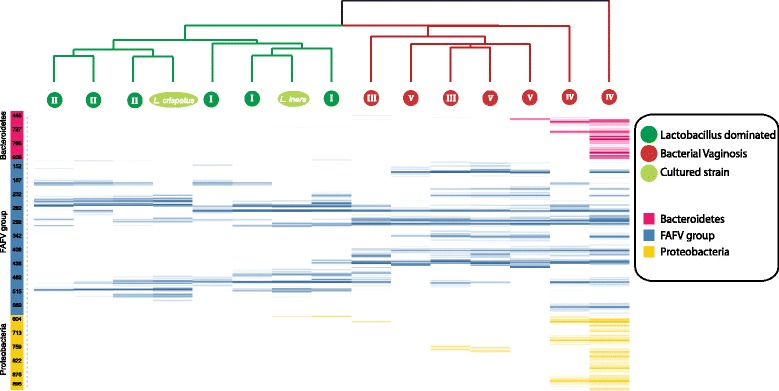
IS-profiling of phyla found in BV negative and BV positive women in relation to cluster group based on 16S rRNA amplicon sequencing data. The abscissa quantifies the cluster of each of 13 samples and 2 cultured strains. In dark green the *Lactobacillus-*dominated samples classified as BV negative are shown, in light green two strains of *L. crispatus* and *L. iners*, and in red the BV positive samples. On the ordinate the phyla are shown, Bacteroidetes (*pink*), FAFV (*blue*), and Proteobacteria (*yellow*). The numbers shown in the ordinate represent the IS-profiling length in the nucleotides. The mean log2 intensity in Relative Fluorescence Units (RFU) is shown by the color intensity as defined by the color bars on the left. FAFV: Firmicutes/Actinobacteria/Fusobacteria/Verrucomicrobia

In Fig. 6, the mean log2 intensity in RFU is shown per phylum for BV positive clusters and BV negative clusters. Firmicutes, including the Family of Lachnospiraceae, and Lactobacillaceae, was the most diverse in the BV positive clusters. An increased diversity of species in BV positive women as compared to BV negative women was found by the use of IS-profiling. Cluster IV included the samples with the highest diversity index.

### *Lactobacillus* abundance and species diversity

We evaluated four molecular indicators to assess BV (Table 3). First, the diversity of the bacterial population, expressed as a number between 0 and 1 by the Gini-Simpson index. Second, the relative abundance of the genus *Lactobacillus*, and third, the relative abundance of *L. crispatus* (both expressed as percentage of the total bacterial population). As a fourth additional *Lactobacillus* marker, we included the relative abundance of *L. iners*, although this bacterium is known to be associated with the BV-negative as well as the BV-positive vaginal microbiota [28]. Diversity of the bacterial population between BV positive and negative samples was well-discriminated by 16S rRNA amplicon sequencing. The average Gini-Simpson diversity index for the BV-positive samples was 0.70 ± 0.15, while the average of BV-negative samples was 0.06 ± 0.04. Although the microarray and to a lesser extent the IS-pro method were not able to discriminate BV on basis of bacterial diversity, we argue that this result does effect the fidelity of this marker, as these methods easily overestimate species diversity from the inclusion of background signals. The dominance of the genus *Lactobacillus* could be assessed by the 16S rRNA sequencing method (0.98 ± .15 vs 0.04 ± 0.04 of BV-negative *vs* BV-positive), but also by both other molecular methods. The presence of *L. crispatus* can be considered the best indicator for the absence of BV, as this bacterium was completely absent (0.00 %) in all samples, confirmed by all three molecular methods. However, this absence did not provide any indication, as the BV-negative cluster I vaginal microbiota profiles do not contain any *L. crispatus*. Our observations confirm that although *L. iners* is in many studies found to be associated with BV, its dominance is also a good indicator for BV-negative samples. The bacterium *L. iners* occured in BV-associated samples only for relative abundances between 0 % and in an exceptional case 32 % of the bacterial population, depending on the detection method used.

## Discussion

### Key findings of this paper

This work confirmed the presence of two clusters of bacterial populations BV negative women, dominated by either *Lactobacillus iners* or *Lactobacillus crispatus* and three distinct clusters in the BV positive women. The cluster profiles in BV positive subjects were relatively high in bacterial species diversity and dominated by a variety of anaerobic species. The Gini-Simpson index of species diversity, and the relative abundance of *Lactobacillus* species appeared consistent indicators for BV. The 16S rRNA amplicon sequencing method was most suitable to assess species diversity, while all three molecular composition profiling methods used in this study were able to indicate *Lactobacillus* abundance in the vaginal microbiota. An affordable and simple molecular test showing a depletion of the genus *Lactobacillus* in combination with an increased species diversity of vaginal microbiota could serve as an alternative diagnostic method for the assessment of BV.

### Universal markers in the BV-associated microbiota

The 16S rRNA amplicon sequence analysis of the vaginal microbiome led to the identification of three clusters of microbiome patterns in Dutch women with BV, and two clusters in women without BV. The latter two clusters resemble two of the major community state types (CSTs), found in US subjects who did not have symptomatic BV, which were dominated by *L. crispatus and L. iners* [28]. However, the present study did not find CSTs of *L. gasseri*, or *L. jensenii,* as observed in other studies [29]*.* All women with BV were deficient in *L. crispatus*. However, *L. iners* was detected in various ratios among women with BV, albeit with lower abundance than BV-negative subjects, as previously confirmed [12].

In women without BV, the vaginal microbiome was dominated by *L. iners* or *L. crispatus* or a mixture of both. *L. crispatus* has been regarded as an important marker for health, yet it is not present in all women deemed healthy [30] and in our study particularly not in the women with cluster 1 microbiome (Table 2). It appears that *L. crispatus* is not solely responsible for maintaining a BV negative state, and it is easily displaced when BV occurs [31]. In BV negative subjects, there was a negative correlation of almost one between *L. crispatus* and *L. iners*. Species of the vaginal microbiome associated with BV in this study include the Family of Coriobacteriaceae (including *A. vaginae*), Leptotrichiaceae (*Sn. sanguinegens* or *L. amnionii*) and of Veillonellaceae (including *D. micraerophilus*). The phyla Bacteroidetes, Actinobacteria, and Fusobacteria, were dominant in BV positive samples. As observed before, unlike the gut [32], the aberrant or disturbed vaginal microbiome is highly diverse, with no systematic occurrence of a single bacterial species shared in the BV-associated microbiota, also evident in our study. Therefore, an overall increased diversity of bacterial species in combination with a depletion of the genus *Lactobacillus* appear good universal molecular markers for diagnosis of BV, in comparison to Nugent scoring, Amsel test or other commercial methods.

### Limitations of this study

With regard to the amplicon sequencing method, the family, genus and species assignment is based on the comparison of specific V5-V7 16S rRNA sequences with those derived from the type strains present in the RDP database. In some cases the obtained V5-V7 16S rRNA amplicon sequence could not be unambiguously assigned to one species (*e.g.* the sequence of *Sneathia sanguinegens* could not be discriminated from that of *Leptotrichia amnionii*). Although the amplicon sequencing methodology appears most accurate in the read-out of species diversity compared to the other methodologies used in this study, also with this method under- or overrepresentation of certain species can occur.

To study the influence of ethnicity on the vaginal microbiota a larger sample size needs to be assessed. Because of financial restrictions, the sample size could not be expanded in this study. However, the findings on bacterial diversity agree with a recent study of samples from Rwandan women [33]. In the present study only BV positive and BV negative women were selected: none with an intermediate Nugent score. A future study could be undertaken to determine if intermediate scores with high diversity actually fall under a BV diagnosis. All the subjects were at high risk of STIs and for that reason attended the clinic for regular check-up. Equating high risk with exposure to STI pathogens is not easy, so the ability to study samples from these subjects on multiple days would be useful to identify if and when exposure occurs and if having BV influences the outcome. A larger sample size is needed to detect correlations between clinical variables, including STI status, VVC, age, or symptoms and signs reported by patients and vaginal microbiota composition.

### Implications for the molecular diagnosis of bacterial vaginosis

For the benefit of clinicians, a simple and inexpensive method is needed to diagnose BV. This could be based on molecular markers identifying women with a vaginal microbiome less resilient to (or at risk for) negative health consequences. A multiplex PCR, which included *L. crispatus*, *L. iners*, and *G. vaginalis*, *A. vaginae*, and *Megasphaera,*has been developed for the diagnosis of BV by Kusters and others [34]. Another has used a *G. vaginalis, A. vaginae*, *Lactobacillus* genus—qPCR tool and found a sensitivity of 93.4 % and specificity of 83.6 % to diagnose BV [35]. Based on these studies and our data of Dutch women, the bacterial diversity and overall abundance of the genus *Lactobacillus* could be considered sufficient as molecular markers to determine BV in the majority of subjects. It remains to be seen if the assessment of BV-clusters with a wide variety of species is significant for the clinical practice, given that there is no consensus about their number and composition.

Another major issue in patient management is that currently no treatments have been developed specifically against any BV-cluster. Formation of an epithelial polymicrobial biofilm with *G. vaginalis* appears to play an important role in BV [36]. Ideally, treatment should be more targeted to destabilize BV biofilms and allow restoration of the subject’s indigenous microbiome associated with health. The failure of industry to develop new treatments leaves patients with sub-optimal care, and with no new therapeutic agents on the horizon, making the best use of current agents, perhaps in combination with (personalized) probiotics, could provide better management of BV for women around the world [37, 38].

## Conclusion

Molecular assessment of species diversity and *Lactobacillus* abundance are useful molecular markers to assess a woman’s BV-status. While diversity can only be accurately assessed by 16S rRNA amplicon sequencing, abundance of the genus *Lactobacillus* can be assessed by all three molecular methods used in this study.

### Ethics approval and consent to participate

The research proposed in this study was evaluated by the ethics review board of the Academic Medical Center (AMC), University of Amsterdam, The Netherlands. According to the review board no additional ethical approval is required for this study, as the samples had been obtained by removal of abundant mucus and are usually discarded as part of the standard procedure for cervical examinations (document reference number W12_086 # 12.17.0104). Clients of the STI clinic are notified that remainders of their samples may be used for scientific research, after anonymization of client clinical data and samples. If clients object, data and samples are discarded. This procedure has been approved by the AMC ethics review board (reference number W15_159 # 15.0193).

### Consent for publication

Clients of the STI clinic are notified that clinical data based on the remainders of their samples may be used for scientific publication, after anonymization. This procedure has been approved by the AMC ethics review board (reference number W15_159 # 15.0193).

### Availability of data and materials

Vaginal sample information and nucleotide-based microarray data (signal to background ratios of fluorescence intensity for each probe) is presented in Additional file 1; the 16S rRNA amplicon sequencing data (DNA sequence, and number of reads) and the family, genus and species identification by the match with the RDP (ribosomal database project) for each 16S rRNA amplicon (V5-V7 region) in Additional file 2 and country of origin of 20 women with BV and 20 women without BV in Additional file 3.

## Additional files

Additional file 1: Vaginal sample information and nucleotide-based microarray data (signal to background ratios of fluorescence intensity for each probe). (XLSX 27 kb) Additional file 2: The 16S rRNA amplicon sequencing data (DNA sequence, and number of reads) and the family, genus and species identification by the match with the RDP (ribosomal database project) for each 16S rRNA amplicon (V5-V7 region). (XLS 2681 kb) Additional file 3: Country of origin of 20 women with BV and 20 women without BV. (PDF 12 kb)

## Abbreviations

BV: bacterial vaginosis
CST: community state type
DGGE: denaturing gradient gel electrophoresis
FAFV: Firmicutes, Actinobacteria, Fusobacteria, and Verrucomicrobia
IS-profiling: intergenic spacer-profiling
NAAT: nucleic acid amplification test
NGS: next generation sequencing
PID: pelvic inflammatory disease
RDP: ribosomal database project.
RFU: relative fluorescence units
STI: sexually transmitted infection
VVC: vulvovaginal candidiasis
