# Supplementary material for: Molecular assessment of bacterial vaginosis by Lactobacillus abundance and species diversity
Source: BMC Infect Dis. 2016 Apr 23;16:180. doi: 10.1186/s12879-016-1513-3 (PMC4841971; doi:10.1186/s12879-016-1513-3)
Supplement: Additional file 3: — Country of origin of 20 women with BV and 20 women without BV. (PDF 12 kb) [file 12879_2016_1513_MOESM3_ESM.pdf]

Table S1. Country of origin of 20 women with bacterial vaginosis and 20 women without bacterial vaginosis.

	BV negative women n = 20	BV positive women n = 20	Total
**Europe**			
Netherlands	13 (32.5%)	7 (17.5%)	20 women (50%)
France	1 (2.5%)		1 woman (2.5%)
Germany	1 (2.5%)		1 woman (2.5%)
Great Britain	1 (2.5%)		1 woman (2.5%)
Turkey	1 (2.5%)		1 woman (2.5%)
**South America**			
Suriname	1 (2.5%)	9 (22.5%)	10 women (25%)
Guyana		1 (2.5%)	1 woman (2.5%)
Colombia		1 (2.5%)	1 woman (2.5%)
Dutch Antilles	1 (2.5%)		1 woman (2.5%)
**Other countries**			
Indonesia		2 (5%)	2 women (5%)
China	1 (2.5%)		1 woman (2.5%)
